# The entry of fetal and amniotic fluid components into the uterine vessel circulation leads to sterile inflammatory processes during parturition

**DOI:** 10.3389/fimmu.2012.00321

**Published:** 2012-10-23

**Authors:** Hiroshi Kobayashi

**Affiliations:** Department of Obstetrics and Gynecology, Nara Medical UniversityKashihara, Nara, Japan

**Keywords:** parturition, inflammation, stretch, amniotic fluid, myometrium

## Abstract

Pro-inflammatory cytokines play an important role during the process of human parturition. The focus of this review was to explore the contribution of biological, biochemical, and genetic changes in the onset of term labor. This article reviews the English-language literature on inflammatory, hormonal, and immunological factors in an effort to identify the molecular basis of human parturition. The majority of the genes and proteins up-regulated in parturition at term are related to four functional categories, mechanical stretch-mediated damage-associated molecular patterns (DAMPs) activation, response to immunity, induction of inflammatory signaling, and progressive uterine myometrial contractility and resultant term birth. Mechanical stretch could promote the entry of amniotic fluid components into the uterine vessel circulation that is the common physiologic mechanism at term prior to labor. The fetal or amniotic fluid-derived DAMPs could activate the immune system. The inflammatory mediators are produced by infiltrating activated leukocytes and by the reproductive tissues themselves such as myometrium, and subsequently lead to uterine contractions. This review supports the sterile inflammation hypothesis that there are at least two phases of human parturition: the initial wave of the entry of amniotic fluid components into uterine vasculatures would be followed by the second big wave of subsequent myometrial contraction.

## INTRODUCTION

Dramatic advances of molecular analysis and biological profiling represent an opportunity to improve an in-depth understanding of human parturition. Parturition is characterized by the activation of innate immune and neuroendocrine mechanisms. Pregnancy is a unique immunological state in which a balance of immune tolerance and suppression may participate in the regulation of the host immune response and protection of the fetus. Oxytocin and corticotropin-releasing hormone are important neuroendocrine pathways involved in parturition (Petraglia et al., 2010). Thus, endocrine–immune interaction controls conditioning of the myometrium and plays as a prelude to the onset of labor.

Furthermore, accumulating evidence suggests that ascending intrauterine infection results in premature birth and high fetal mortality (Martius and Eschenbach, 1990). There has been an increased awareness of the role of infection and inflammation at the time of parturition. Bacterial infection and sterile inflammation (a physiological process) are key mechanisms of human preterm and term labor, respectively. Several studies focused on the feedforward loop in which, near the end of pregnancy, the pro-inflammatory cytokine–prostaglandin (PG) axis activates the uterus (Golightly et al., 2011). Excessive inflammation produces uterine contractile stimulants leading to labor and delivery.

Despite considerable research and progress in the technology of reproduction, the causes of the initial events driving parturition remain obscure. We review the contemporary literature on sterile inflammation that support mechanism of parturition at term.

## STUDY METHODOLOGY

The present study reviews the literature for biological studies of human parturition. Data pertaining to *in vitro* and *in vivo* studies were included. A computerized literature search was performed to identify relevant studies reported in the English language. All abstracts from Medline electronic database were reviewed to identify papers for full-text review. The web-based database were searched, combining the keywords “genome-wide,” “proteomics,” “onset,” “labor,” “term,” “myometrium,” “cervix,” “amniotic fluid,” “TLR,” “inflammation,” “immunity” “leukocytes,” “cytokine,” “complement,” and “NF-kappaB” with “parturition.” Additionally, references in each study were searched to identify potentially missed studies. Target publications are mainly reports on human studies and animal models, as well as basic studies in gene and protein expression systems. Abstracts were not included, since they do not undergo a stringent peer review process.

## PARTURITION AFFECTED BY THE STATUS OF IMMUNITY

Alterations in maternal immunity, peripheral tolerance and feto-maternal tolerance of uteroplacental unit have been seen during pregnancy, the so-called “immunological paradox” (Spencer et al., 2008). In humans, there are key players in the regulation of the pathway involved in suppression of immune responses. NK cells, T regulatory (Treg) cells, the Th1/Th2 shift and complement system had a key role in the suppression of immune responses (Kokubu et al., 2005; Guerin et al., 2009). NK cells were critical for the success of pregnancy and closely involved in parturition (Kokubu et al., 2005). Treg cells favored fetal development and escaped from the host immune system through suppression of the activation of immune cells including antigen presenting cells, CD4^+^ and CD8^+^ T lymphocytes. Treg cell numbers were considered to increase early in pregnancy and then began to decline at parturition and decreased in the postpartum period (Norris et al., 2011). These specific immune systems might be necessary to achieve maternal alloantigen tolerance during pregnancy. This phenomenon is referred to as the Th1/Th2 shift. Down-regulation of the Th1 response and Th2 predominance were associated with successful pregnancy maintenance (Sykes et al., 2012). Th1 cells secreted pro-inflammatory cytokines such as interferon-gamma (IFN-γ) and tumor necrosis factor-alpha (TNF-α). The Th1 cytokine-mediated Toll-like receptor (TLR) activation contributed to the inflammatory response in the initiation of labor.

In contrast, pregnancy hormones such as progesterone, estradiol, leukemia inhibitory factor, and PGD_2_ promoted the Th2 cell profiling by modulating a direct tie between hormone and immune function. Th2 cells produced interleukin (IL)-4, IL-5, IL-13, and IL-10. IL-10 is an anti-inflammatory cytokine produced primarily by decidual macrophages and overexpressed at the maternal–fetal interface and is crucial for dampening inflammation. In animal experiments using rhesus monkeys, this cytokine blocked IL-1β-induced preterm labor (Sadowsky et al., 2003). IL-10 also inhibited IL-1β-induced decreases in placental PG dehydrogenase (PGDH) expression (Pomini et al., 1999). There was a dynamic spatial and temporal expression pattern for IL-10 in human placenta: IL-10 was expressed in the first and second trimester placental tissues. In term tissue, however, this cytokine diminished before the onset of labor (Hanna et al., 2000). IL-10 down-regulated the expression of Th1 pro-inflammatory cytokines through suppression of activation of the transcription factor nuclear factor-kappaB (NF-κB), a driver gene for inflammation-related signaling pathway. These data support the requirement of the Th1/Th2 shift for successful parturition.

In addition, one of the initial responses of this innate immunity may be an activation of the complement cascade. Complement can generate biologically active products, which trigger inflammation. Normal pregnancy was characterized by an increase in anaphylatoxin C5a in the maternal circulation in the third trimester, suggesting that the complement system is activated. C5a up-regulated pro-inflammatory and pro-labor mediators, including pro-inflammatory cytokines (IL-6 and IL-8), cyclooxygenase (COX)-2, PGE_2_ and PGF_2_α, matrix metalloproteinase (MMP)-9, and 8-isoprostane in human gestational tissues via the C5a receptor (CD88)-mediated NF-κB activation (Lappas et al., 2012). Abundant research has demonstrated complement activation in an innate immunity of human parturition (Gallery et al., 1981; Benson et al., 2006; Benson, 2007; Soto et al., 2009; Gonzalez et al., 2011; Kato et al., 2012; Lappas et al., 2012), but has yet to investigate whether complement activation is the result of fetal antigen leaking into the maternal circulation.

## MECHANICAL STRETCH OF UTERINE MYOMETRIUM AT TERM

Many investigators have analyzed genome-wide transcriptomes and proteomics of the reproductive tissues at different stages of parturition. At term, uterine myometrium, including myometrial smooth muscle cells and fibroblasts, was stretched by growing fetuses. Molecular mechanism mediating stretch-induced signaling pathways has been elucidated. Cyclic mechanical stretch induced an increase in secretion of pro-inflammatory cytokines in myometrial smooth muscle cells compared to non-stretch controls (Sooranna et al., 2004; Kendal-Wright et al., 2010; Hua et al., 2012). This increase in cytokine production correlated with activation of NF-κB (Mendelson, 2009). Mechanical stretch also stimulated COX-2 expression through activation of the activated protein (AP)-1 system (Sooranna et al., 2004). Thus, cyclic stretch and release in myometrial smooth muscle cells stimulated a robust activation of NF-κB and AP-1. Some of the important genes up-regulated in human myometrium during term labor were monocyte chemotactic protein-1 (MCP-1, also known as C-C chemokine motif ligand 2, CCL-2), IL-8, and TNF-α. MCP-1 was a member of a large chemokine family and displayed chemotactic activity for monocytes/macrophages, as well as promoting macrophage activation (Esplin et al., 2005). MCP-1 expression was enhanced by mechanical stretch of the uterine myometrium. IL-8 and TNF-α, whose expression was specifically restricted to myometrium after the onset of labor, were potential candidates of contraction-associated cytokines. In contrast, both IL-1β and IL-6 were present in term myometrium before and during labor, suggesting that these cytokines were involved in the preparation or conditioning for the synchronized contractions of labor (Sehringer et al., 2000). IL-1β participated in the regulation of the myometrial contractions via an increase in PGs production (Hertelendy et al., 1993). IL-1β was synthesized as an inactive precursor, pro-IL-1β, and then cleaved into the active form through cytosolic protein complexes termed “inflammasomes” (Gotsch et al., 2008). The placenta expressed the inflammasomes. Cellular stress in response to inflammatory conditions accounted for activation of the inflammasomes, which occurred during labor. The previous elegant review discussed the role of the inflammasomes system and their potential to contribute to the pathogenesis of preterm birth (Abrahams, 2011). Unfortunately, we have very little understanding of their function in normal pregnancy and the onset of term labor.

These data suggest that transduction of the stretch signal in myometrial smooth muscle cells involves alteration of the gene expression signature. Activation of NF-κB and AP-1 increased expression of several genes implicated in the control of immunity and inflammation (Mendelson, 2009; Khanjani et al., 2011). MCP-1 locally mediated leukocyte migration into uterine myometrial tissues. Myometrial smooth muscle cells can play a role as immune cells and participate in the sterile inflammation at term (Khanjani et al., 2011; Shynlova et al., 2012). Taken together, mechanical stretch-induced NF-κB/AP-1 activation, which occurs prior to labor, modulates the expression of numerous inflammation-associated genes that are directly or indirectly involved in the positive feedback loop during parturition.

## THE ENTRY OF FETAL AND AMNIOTIC FLUID COMPONENTS INTO THE UTERINE VESSEL CIRCULATION

Prior to labor, there were prominent changes in the myometrial fibers that increase the distance between muscle layers and promoted edema. These cells exhibited such morphology as shearing, shrinkage, and apoptosis. Endothelial cell damage in the uterine myometrium were very common at term prior to labor. The vascular lumen of endothelial cells contained fibrin and platelet thrombi, microparticles, desquamated endothelial cells, amniotic squamous cells, and mucoid material (Leong et al., 2008). The entry of amniotic fluid components into the uterine vessel circulation might be the common physiologic mechanism. Histologically, these changes were present in myometrial tissues obtained during labor at term, providing a mechanism by which fetal and amniotic fluid components may access myometrial cells (Leong et al., 2008). In addition, small amount of fetal red cells were normally detectable in peripheral blood of the mother in all pregnancies, indicating that fetal cells can enter the maternal circulation (Ahmed and Abdullatif, 2011). The presence of not only intact fetal cells but also fetal-origin nucleic acids (cell-free fetal DNA and RNA) in maternal blood has been identified. Cell-free fetal nucleic acids afford the opportunity for the promising prenatal genetic testing. Part of the fetal DNA fragments derived from the placenta. These data support that a substantial amount of fetal antigens might be transported to the uterine vasculature and maternal circulation at term prior to labor.

Changes in the recognition and adaptation to a set of foreign antigens would be a mechanism of the onset of labor. The maternal responses to an alloantigen challenge were reduced during pregnancy (Spencer et al., 2008), while, alloantigens resulted in immune-mediated fetal rejection in the term parturition. Recently, pattern recognition receptors (PRRs) responsive to unique molecules, termed pathogen-associated molecular patterns (PAMPs), have received considerable attention as possible contributors to the onset of preterm labor. Microorganisms have PAMPs that were recognized by PRRs such as TLRs, Nod-like receptors (NLRs), and the inflammasomes (Tang et al., 2012). PRRs recognized not only PAMPs, but also host-derived danger signals “alarmin” or damage-associated molecular patterns (DAMPs) derived from damaged tissue. In general, DAMPs are known to be cell-derived immunity. The fetal DNA found in the maternal circulation could act as DAMPs through PRRs such as TLR9 or AIM2 (absent in melanoma 2; Barber, 2011). The TLR9 has an ability to bind structurally highly conserved microbial molecules such as CpG motif-containing DNA and subsequently initiates the production of Th1 pro-inflammatory cytokines and chemokines (Barber, 2011). AIM2 acts as a DNA sensor in innate immunity and mediates inflammatory responses involving IL-1β. AIM2 also triggered the assembly of the inflammasomes. Cell-free fetal DNA would mediate innate immune signaling that provides an important step toward initiation of parturition.

Furthermore, hyaluronan, a component of the extracellular matrix, was a component of the DAMPs associated with NLRs. Intra-amniotic hyaluronan levels were elevated in pregnancies. Hyaluronan was released into the extracellular milieu and also amniotic fluid where it modulates immune activity. Yet, this hypothesis has not been proved.

These initial events prior to labor hint at a possible causative role. During pregnancy and prior to labor, women were tolerant of their semi-allogeneic fetal components: the maternal immune system came into contact with trophoblasts and other semi-allogeneic components, including amniotic fluid, fetal cells, and cell-free fetal DNA. The modulation of cell-mediated immunity caused by a substantial amount of DAMPs at term prior to labor may be responsible for the increased susceptibility to parturition.

## INFILTRATION OF LEUKOCYTES IN UTERINE MYOMETRIUM AND CERVIX

An accumulating body of evidence has demonstrated that uterine myometrial contraction coincident with the onset of term labor was accompanied by the massive influx of leukocytes in all regions of uterine myometrium, amnion, choriodecidua, and cervix following spontaneous labor compared with non-laboring tissues (Thomson et al., 1999; Keski-Nisula et al., 2000; Osman et al., 2006; Gomez-Lopez et al., 2011; Hamilton et al., 2012; Shynlova et al., 2012). Histological analysis demonstrated that, in the myometrium, tissue macrophages, neutrophils, and T lymphocytes massively increased coincident with the onset of labor at term (Thomson et al., 1999; Keski-Nisula et al., 2003; Osman et al., 2006). Marked myometrial inflammation was not associated with the prediction of pathological conditions such as infection (Keski-Nisula et al., 2003). The influx of fetal leukocytes into the myometrium has been implicated in the initiation of parturition in mice (Kim et al., 2006). During human labor, however, fetal macrophages from the amniotic cavity or the chorioamniotic membranes did not migrate into the myometrium (Kim et al., 2006). Leukocytes into the myometrium was a maternal origin. The uterus at term was infiltrated with inflammatory cells, which was subsequently associated with advanced labor and uterine contraction, because pro-inflammatory cytokines such as TNF-α can stimulate uterine smooth muscle cell contractility (Keski-Nisula et al., 2003; Yellon et al., 2003; Fitzgibbon et al., 2009; Kamel, 2010; Lee et al., 2012). Inflammatory cells orchestrate processes required for initiation of the myometrial contraction (Thomson et al., 1999; Osman et al., 2003).

The uterine cervix must be disorganized before, during and after parturition, via release of proteolytic enzymes and followed by a tissue repair postpartum. Leukocyte density increased two- to threefold between the first trimester of pregnancy and term, prior to the onset of labor (Spencer et al., 2008). A marked increase in macrophage density was observed after the onset of labor (Keski-Nisula et al., 2000; Timmons et al., 2009; Kamel, 2010). This occurred during the course of cervical softening and effacement (Bokström et al., 1997). In contrast, neutrophils specifically increased in the postpartum period and were involved in the postpartum tissue repair (Thomson et al., 1999; Hamilton et al., 2012).

Taken together, leukocyte infiltration is a complex process involving at least two steps, including the first step, mechanical stretch of uterine myometrium without involvement of resident macrophages, and then the second step, the entry of fetal and amniotic fluid alloantigens into the maternal circulation. These steps might drive myometrial chemokine expression primarily via activation of NF-κB, which in turn results in a prominent leukocyte infiltration into the uterus. DAMPs such as fetal antigens activated infiltrated macrophages via the TLR/AIM2/NF-κB pathway. The infiltration of myometrium (before the onset of labor) and cervix (in the postpartum) with activated leukocytes has been associated with the initiation of parturition and a rapid repair postpartum, respectively.

## INFLAMMATORY GENE REGULATORY NETWORKS IN PARTURITION

Several investigators used a network mining algorithm to identify tightly connected gene expression pathways that were frequently present in microarray data sets samples. Global gene expression analyses and single gene approaches revealed that human labor involved the infiltration of specific leukocyte subsets and the secretion of autocrine and paracrine mediators, including NF-κB, pro-inflammatory cytokines (IL-1, IL-6, TNF-α), chemokines (IL-8/CXCL-8, MCP-1/CCL-2), IL-1 receptor accessory protein (IL-1RAP), TLRs, COX-2, contraction-associated proteins (CAPS), oxytocin receptor (OXTR), connexin-43 (CX-43), the prostaglandin F receptor (FP), hypoxia-inducible factor (HIF), thrombospondin 1 (TSP-1), MMP-2, and MMP-9 (Vora et al., 2010; Li et al., 2011; Lim et al., 2012). The majority of these genes were downstream targets of NF-κB and PRRs such as TLRs. Spontaneous term labor group is associated with increasing activation of the NF-κB signaling network relative to the term no labor cohort (Vora et al., 2010). Therefore, sterile inflammation has underlied parturition at term and the TLR/NF-κB axis in macrophages is an essential pathway.

## ENDOCRINE AND PROSTANOID PATHWAYS

Myometrial contractility has been the predominant focus for the mechanism that contributes to regulation of development of parturition and initiates labor. The endocrine status affects the process of parturition. The potential factors included PGs, oxytocin, nitric oxide, COX-2, cytokines, as well as endocrine mediators such as estrogen, progesterone, corticotrophin releasing hormone, and cortisol. Activation of contractile genes (e.g., COX-2, OXTR) was directly promoted by transcription factors NF-κB and AP-1. Such changes in prostanoids and COX-2 pathways seem to be inflammation-mediated physiological responses at the later stages of parturition. It is likely that all these factors were involved in the feedforward loop during parturition (Christiaens et al., 2008; Golightly et al., 2011).

## SUMMARY

**FIGURE 1:**
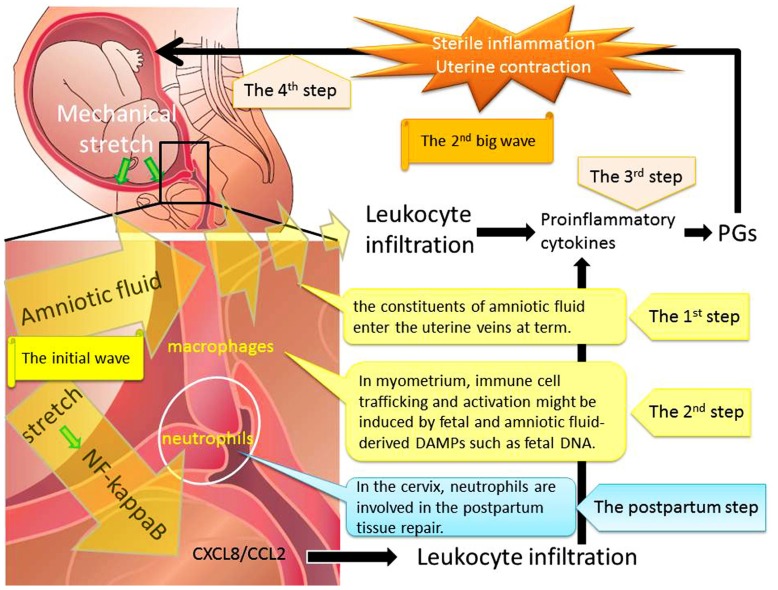
**Mechanical stretch and subsequent leukocyte infiltration are essential for labor onset at term.** *Step 1*: Mechanical stretch synergistically augmented leukocyte infiltration and production of pro-inflammatory chemokines via the NF-κB pathway. *Step 2*: The entry of amniotic fluid components into uterine vasculatures promoted myometrial infiltration with macrophages and neutrophils. *Step 3*: Infiltrating leukocytes could induce expression of pro-inflammatory cytokines and PGs through the activation of NF-κB. *Step 4*: The final step is the progressive uterine contractility and resultant term birth. We hypothesized that the initial wave of mechanical stretch and leukocyte infiltration would be followed by the second big wave of sterile inflammation and subsequent myometrial contraction.

This review focuses on the contribution of biological, biochemical, and genetic changes during each phase of activation in the process of parturition. This process consists of four steps (**Figure 1**). The first step is mechanical stretch of myometrium which can promote the entry of amniotic fluid components into the uterine vessel circulation at term prior to labor. The constituents of amniotic fluid include fetal and amniotic fluid-derived DAMPs such as cell-free fetal DNA and RNA. The second step consists of immune cells trafficking and activation which might be induced by fetal and amniotic fluid components. The third step is inflammatory reactions with the release of Th1 cytokines and down-regulation of the Th2 response. This seemingly irreversible step consists of the enhanced production of PGs and endocrine mediators. The final step is the progressive uterine contractility and resultant term birth. Therefore, immune cell trafficking and activation induced through myometrial stretch-mediated DAMP activation are part of the initial mechanism that immediately enhances uterine myometrial contractility and initiates parturition.

In conclusion, there are at least two phases of human parturition: the initial wave of the entry of amniotic fluid components into uterine vasculatures would be followed by the second big wave of sterile inflammation by infiltrating leukocytes and subsequent myometrial contraction.

## Conflict of Interest Statement

The author declares that the research was conducted in the absence of any commercial or financial relationships that could be construed as a potential conflict of interest.
